# Hospitals in Native Regiments

**Published:** 1919-05-03

**Authors:** 


### Hospitals in Native Regiments.

The Views of Indian Soldiers from the Front.

English nurses at the Front have testified to the lamentations of wounded French soldiers when being transferred from hospitals regulated by a carefully trained British staff to others where they knew they would be attended to by their well-meaning but inadequately experienced countrywomen. In his recent work "Indian Studies," General Sir O'Moore Creagh predicts an unfavourable contrast with the Indian hospitals in native regiments. He writes: "The hospitals in native regiments were long a disgrace to civilisation. They were managed on old and obsolete methods. There was no system of dieting, the sick fed themselves on the ordinary ration; there was no provision of hospital clothing, or of decent bedding for the sick Indian soldier. There was no operating-theatre; there was no room for segregating infectious cases. The only way this could be done was either to screen off a part of the one long room of which the hospital consisted, or to put such cases outside in all weathers in single fly-tents. The hospital was like a British railway station cowshed. Medical instruments were deficient, medical comforts were non-existent. During my whole service the same had been the case."

After particularising his Government's hampered attempts at remedy when Commander-in-Chief in 1912, the author adds: "What a contrast the Indian soldiers who have been in English hospitals will find when they return home if the state of affairs I left has not been altered; how they will compare notes with their comrades in India!"

